# Anorexia and attachment: dysregulated defense and pathological mourning

**DOI:** 10.3389/fpsyg.2014.01218

**Published:** 2014-10-28

**Authors:** Elisa Delvecchio, Daniela Di Riso, Silvia Salcuni, Adriana Lis, Carol George

**Affiliations:** ^1^Dipartimento di Psicologia dello Sviluppo e della Socializzazione, Università di PadovaPadua, Italy; ^2^Department of Psychology, Mills CollegeOakland, CA, USA

**Keywords:** attachment, adult attachment projective picture system, anorexia nervosa, defense mechanisms, dysregulation

## Abstract

The role of defensive exclusion (Deactivation and Segregated Systems) in the development of early relationships and related to subsequent manifestations of symptoms of eating disorders was assessed using the Adult Attachment Projective Picture System (AAP). Fifty-one DSM-IV diagnosed women with anorexia participated in the study. Anorexic patients were primarily classified as dismissing or unresolved. Quantitative and qualitative analyses of defensive exclusion were carried out. Results showed potential benefits of using the AAP defense exclusion coding system, in addition to the main attachment classifications, in order to better understand the developmental issues involved in anorexia. Discussion concerned the processes, such as pathological mourning, that may underlie the associations between dismissing and unresolved attachment and anorexia. Implications for developmental research and clinical nosology are discussed.

## INTRODUCTION

Anorexia nervosa (AN) is a severe form of psychopathology which is difficult to treat (Steiner et al., 2003). Symptoms include refusal of maintaining a minimal body weight according to age and height (less than 85% of the expected), intense fear of gaining weight or becoming fat (even when the subject is underweight), exaggerated weight concerns, desperate attempts to prevent weight gain through self-induced vomiting, laxative abuse, or excessive dieting and exercise, and denial of the seriousness of current low body weight or wrong perception of body weight (dysmorphophobia; American Psychiatric Association [APA], 1994). The anorexic identity appears to be one of the most dangerous clinical conditions and has severe psychological implications (Fairburn and Brownell, 2002). It shows a high incidence of comorbid diagnoses and the highest mortality rate among all mental disorders (Attia, 2010). It is 10 times more frequent in females than in males (Woodside et al., 2001), and the highest incidence occurs in adolescent girls and young women (0.5–1%; American Psychiatric Association [APA], 1994).

Researchers have identified biological, cognitive, family-related, and socio-cultural factors that contribute to and maintain eating disorders (e.g., Bruch, 1973; Kiang and Harter, 2006; Farber, 2008). Steiner et al. (2003) emphasized that a developmental psychopathological approach is needed to unify these different perspectives. They suggested that identifying developmental risks and buffering factors was essential to address etiology and treatment. One important developmental factor is attachment, intended as the foundation of emotional- and self-regulation. It concerns beliefs about the accessibility and worthiness of self and others, and capacities to review and reflect on interpersonal experience (e.g., Cassidy and Shaver, 2008; Zachrisson and Skårderud, 2010). Attachment insecurity is an established risk factor for eating disorders in general and, more specifically, for anorexia (e.g., Pierrehumbert et al., 2002; Troisi et al., 2005, 2006; Canetti et al., 2008; Dozier et al., 2008; Tereno et al., 2008; Zachrisson and Skårderud, 2010; Amianto et al., 2011). The prevalence of insecure attachment in eating disorders is estimated between 70 and 100% (Ramacciotti et al., 2001; Zachrisson and Kulbotten, 2006). So far, researchers approached the study of eating disorders using two different research methodologies. Both of them are connected to attachment research, but differ as regards conceptual histories and forms of assessment (Waters et al., 2002; Crowell et al., 2008).

The majority of eating disorder studies was conducted following the romantic adult attachment perspective. This approach conceives attachment as a personality style related to satisfaction in romantic love and in adult relationships (i.e., marriage and dating; Hazan and Shaver, 1987; Kenny and Hart, 1992; Friedberg and Lyddon, 1996; Chassler, 1997; Sharpe et al., 1998; Ward et al., 2000; Broberg et al., 2001; Latzer et al., 2002; Orzolek-Kronner, 2002; Pierrehumbert et al., 2002; Tasca et al., 2004, 2006a,b; Evans and Wertheim, 2005; Troisi et al., 2005, 2006; Kiang and Harter, 2006; Eggert et al., 2007). In this perspective, attachment style was assessed using self-report questionnaires that identified secure, avoidant, ambivalent, and for some measures, fearful attachment styles. According to this perspective, insecure attachment style is significantly associated with eating disorders. However, results were mixed as to which particular insecure attachment style was the most prominent (e.g., Elgin and Pritchard, 2006; Kiang and Harter, 2006; Tasca et al., 2007; Tereno et al., 2008).

In the field of eating disorders, there are relatively few adult attachment studies adopting a developmental approach. Such approach evaluates an individual’s state of mind on the basis of experiences with parental attachment figures during childhood. To our knowledge, all studies to date used a single assessment methodology, the Adult Attachment Interview (AAI; George et al., 1984, George et al., unpublished manuscript; Main et al., unpublished manuscript; Hesse, 2008). The AAI designates five adult attachment patterns – secure, dismissing, preoccupied, unresolved, and cannot classify. This last pattern is associated with abuse or other severe attachment threats (Hesse, 2008). According to this perspective, most of the existing studies reported a predominance of dismissing and unresolved attachment (Cole-Detke and Kobak, 1996; Fonagy et al., 1996; Ward et al., 2001; Ringer and Crittenden, 2007; Barone and Guiducci, 2009), with the exception of one study that reported a prevalence of dismissing and preoccupied attachment (Ramacciotti et al., 2001).

Establishing the distribution of attachment classifications has been the standard first step in investigating attachment correlates in psychopathology (Dozier et al., 2008). The predominance of dismissing and unresolved adult attachment and analogous personality style groups (avoidant, fearful) in eating disorder samples is striking, especially for anorexia. Distributions are different from those established for other psychopathological conditions or for control samples (van IJzendoorn and Bakermans-Kranenburg, 1996; Bakermans-Kranenburg and van IJzendoorn, 2009; Cassibba et al., 2013). However, distribution patterns do not adequately explain the underlying processes that contribute to the intense separation anxiety, fear of abandonment, and maladaptive behavior which are associated with this specific eating disorder. Several studies reported experience and state of mind rating scales more in detail, exploring different dynamics even in the same classification patterns, in an effort to better understand these processes.

From a developmental perspective, both behavioral control of attachment figures and dysregulated frightened states of mind define the disorganized attachment pattern (Main and Solomon, 1990; Solomon et al., 1995; Lyons-Ruth and Jacobvitz, 2008; Marvin and Britner, 2008). Unresolved attachment (unresolved for loss and abuse) is conceived as the adult analog of children’s disorganized attachment pattern (Hesse, 2008). The prevalence of unresolved attachment in the eating disorder literature suggests that disorganization plays a strong etiological role. Solomon and George (2011, p. 4) argued that disorganization is not limited to behavior and broadly “represents dysregulation of co-adaptive processes (in attachment-caregiving relationships) at the level of behavior, physiology, and representation.”

George and West (2012) argued that the predominant theory regarding disorganization in adulthood, although fruitful, needed to be updated, and primarily based on the model of attachment dysregulation. Many researchers reported the AAI approach as limiting in the study of risk factors (Schuengel and van Ijzendoorn, 2001; Steele, 2003; Hesse, 2008; Buchheim and George, 2011). In an attempt to broaden the conceptualization of trauma beyond loss and abuse (the only risk experience assessed by the AAI), George and West (2012) defined *attachment trauma* as to include all the experiences which represent a threat to personal safety and to the integrity of attachment relationships. This view would include a broader range of severe dysregulation risk factors, such as family experiences (e.g., parental conflict, even if not directed toward the child), alcoholism, and psychopathology (e.g., Solomon and George, 2011), community terrorism and violence (e.g., Almqvist and Broberg, 2003; De Haene et al., 2010), and institutionalization (e.g., Schuengel and van Ijzendoorn, 2001). George and West (2012) emphasized that all forms of attachment trauma constitute a *loss*, which, if not mourned, would lead to “the persistent and insatiable nature of the yearning for the *lost* attachment figure” (Bowlby, 1980, p. 26 – italics added) and to the dysregulation associated with feelings of fear, rage, helplessness, abandonment, and isolation (George and West, 2012)

Mourning requires re-organizing and integrating representations of self and attachment figures to match the reality of their inaccessibility (Bowlby, 1980; West and Sheldon-Keller, 1994). When mourning is complete, the individual accepts experience as truthful and permanent, and dismantles and transforms perceptions of the frightened, unprotected, and helpless self to begin anew (Bowlby, 1980; West and Sheldon-Keller, 1994; George and West, 2012). When mourning is incomplete, representational models of the self and attachment figures remain unchanged and persist (Bowlby, 1980; George and West, 2012). In this process, defenses play a central role (Bowlby, 1980). They refer to automatic and unconscious attention processes which select, exclude, and transform behavior, thought, and emotions so as to modulate attachment distress and prevent psychological breakdown. Adaptive use of defensive processes promotes healthy mourning, whereas maladaptive dependence on defenses compromises relatedness, incrementing the risk for “breakdowns in functioning.” Such breakdowns, in turn, undermine the ability to cope with distress (Bowlby, 1980, p. 72). Bowlby (1980) defined three forms of defense: *deactivation, cognitive disconnection*, and *segregated systems*. Deactivation encompasses strategies that shift attention away from attachment events, memories, or feelings. Deactivation is associated with evaluations of self and others as not deserving care and with the rejection or neutralization of attachment needs (Solomon et al., 1995; George and Solomon, 2008). Normative forms of deactivation are the main organizing defense that defines the insecure-avoidant pattern in children and the dismissing attachment in adults (Solomon et al., 1995; George and West, 2001). Maladaptive forms of deactivation foster a form of pathological mourning which Bowlby (1980) called “failed mourning” or “prolonged absence of conscious grieving” (George and West, 2012).

Cognitive disconnection literally disconnects attachment distress from its source, undermining consistency, and the ability of holding in mind a unitary view of events and emotions which follow attachment activation (Bowlby, 1980). Disconnection results in confused evaluations of self and others that make it difficult to turn away from attachment (i.e., deactivate; Solomon et al., 1995; George and West, 2001). Normative forms of cognitive disconnection are the main organizing defense that defines ambivalent-resistant attachment in children and preoccupied attachment in adults (Solomon et al., 1995; George and West, 2001). This defensive process is highly present in most individuals and can be seen as a background “mental noise” that remains active in all individuals. However, individuals classified as preoccupied do not manage to keep such noise in the background and are therefore overwhelmed by this “mental noise.” Maladaptive forms of cognitive disconnection are associated with a form of pathological mourning that Bowlby (1980) described as “preoccupation with personal reactions and sufferings.” It refers to a chronic mourning state characterized by disorganized behavior pining, anger and endless search for attachment figures. Attachment state of mind is akin to “living in the war zone” and manifests on the AAI as a subgroup of the preoccupied classification, preoccupied with trauma (George and West, 2012).

Segregated systems encompass painful and threatening attachment experiences and affects which are blocked from consciousness, such as those associated with unbearable loss (Bowlby, 1980). Segregated systems can dysregulate attachment under conditions of stress or threat and are associated with behavioral and psychological hypervigilance (i.e., a fear response). Such hypervigilance manifests itself either via loss of control or constricted (i.e., frozen) forms (Solomon et al., 1995; George and West, 2001). Segregated systems are the building blocks of mourning (Bowlby, 1980). When experience and affect are modulated, transformed, and integrated they are what Bowlby considered as the residual “scars” of loss. On the contrary, when normative defense breaks down, dysregulated experience and affect define a disorganized form of chronic mourning (Bowlby, 1980). Dysregulated segregated systems are the underlying processes associated with disorganized attachment in children and with unresolved attachment on the AAI in adults (Solomon et al., 1995; George and West, 2001; George and Solomon, 2008).

In the present study we propose that defensive processes linked to attachment trauma and associated with maladaptive deactivation and pathological mourning may explain to some intent severe manifestations of eating disorders. More specifically, anorexia presents a distinct pattern of symptoms associated with a lack of attention or ability to discriminate inner feelings and distress cues, with the illusion of having control over feelings of emptiness, overwhelm, and fear (e.g., Bruch, 1973; Leon et al., 1993; Eggert et al., 2007). As previously mentioned, Fonagy et al. (1996) reported that 93% of their eating disorder patients were unresolved. In Ward et al.’s (2001) study, only 35% of their anorexic sample was unresolved, however, 80% was assigned to a classification indicative of some form of dismissing/devaluing attachment (irrespective if unresolved or not). These patterns suggest that deactivating defenses shift attention away from personal distress. However, when fragile in the face of trauma, defensive breakdown can lead to dysregulation (i.e., unresolved).

The present study was designed to address the issue of attachment and defensive exclusion patterns (deactivation and segregated systems) in young adult women diagnosed with DSM-IV anorexia. In order to accomplish this goal, we used the *Adult Attachment Projective Picture System* (AAP), the only adult attachment measure that (1) provides AAI-based classifications, (2) evaluates defensive processing patterns, (3) makes a distinction between “normative” and “traumatic” segregated systems and (4) addresses pathological mourning, as assessed by three or more traumatic indicators of segregated systems in the same story.

Specifically, in light of these considerations, this study had the general aim to investigate patterns of attachment as rated in the main classification of the AAP coding system, examining the role of two typical forms of defensive exclusion in order to assess the dynamics underneath the attachment profile. Moreover, according to the pathological mourning perspective, specific forms of pathological mourning in the anorexia sample were explored.

Four sets of hypotheses were considered in the current work:

(1) It was expected the majority of anorexic patients to show an unresolved or dismissing attachment pattern (Ramacciotti et al., 2001; Zachrisson and Kulbotten, 2006).
(2) Differences were expected between the current sample and the non-clinical sample of similar age from van IJzendoorn and Bakermans-Kranenburg (1996), and from Cassibba et al.’s (2013) data on attachment classifications measured by the AAI (i.e., adolescents and young adults). Furthermore, anorexic patients’ distribution of attachment classifications was expected to be significantly different from clinical samples (van IJzendoorn and Bakermans-Kranenburg, 1996; Cassibba et al., 2013), with a higher percentage of unresolved and dismissing patterns in the current sample.
(3) A more frequent use of traumatic segregated systems than normative ones was expected in the stories.
(4) The fourth hypothesis predicted that attachment in anorexia would be associated with pathological mourning. More specifically, high percentages of Ds profiles with failed mourning and U profiles with chronic mourning were expected. As a consequence, we expected that anorexia would not be associated with the form of pathological mourning linked to chronic preoccupation with trauma (Buchheim and George, 2011; George and West, 2001, 2012).

## MATERIALS AND METHODS

### PARTICIPANTS

Participants were 51 female patients with anorexia (AN) recruited in a mental health assessment and treatment center in Italy. Age in the sample ranged from 18 to 36 years (*M* = 24.82; SD = 4.91). Patients were diagnosed according to DSM-IV criteria (American Psychiatric Association [APA], 1994) by expert psychiatrists. Thirteen (26%) patients had co-morbid diagnoses: obsessive–compulsive disorder (12%), major depressive disorder (8%), and borderline personality disorder (6%). The mean age at the onset of anorexia was 16.5 years. Forty seven (92.5%) were single and three (7.5%) were married. Thirty one (77.5%) were full-time university students, seven (17.5%) were employed, and two (5%) were unemployed. The socioeconomic level of the sample was medium to high. Two-thirds of the sample came from intact families. Although the participants’ mothers were well educated, the majority of them (63%) did not work outside their home.

These demographics indicated that the patients from the current sample were similar to those in the majority of eating disorder studies with regard to socioeconomic status and educational level. The external sociological variables indicated that patients had a stable family constellation and considered their mother as their primary caregiver.

### PROCEDURE

Attachment assessment was done prior to treatment; none of the participants was in any kind of psychotherapeutic treatment at the time of the study. The patients gave their informed consent to participate in the study. There were no dropouts from AAP testing.

### MEASURES

The AAP (George et al., unpublished manuscript; George and West, 2001, 2012) is a free-response assessment of adult attachment based on the analysis of the “story” responses to a set of theoretically derived attachment-related drawings. The AAP stimulus set includes eight stimuli, a warm-up picture and seven attachment scenes ordered to progress from mildly to highly distressing situations (Buchheim et al., 2006). Participants are instructed to tell a story for each stimulus. The story elements include a description of how the situation began, the characters’ actions, feelings, and thoughts, and how the situation ends. Each attachment stimulus response is coded for story content (agency, connectedness, and synchrony), defensive processes (deactivation, cognitive disconnection, segregated systems) and the inclusion of personal experience. Coding is completed using verbatim transcripts of audiotaped AAP responses. Attachment group classification is determined based on the patterns of content, defense, and personal experience codes demonstrated across the entire set of seven attachment stories. The AAP identifies the four standard adult attachment patterns: three organized patterns (secure-F, dismissing-Ds, preoccupied-E), and one disorganized pattern, named unresolved-U. For a description of AAP coding variables see George and West (2012). For the purpose of this paper, a more specific explanation of defensive processes will be provided. Deactivation attempts to shift attention away from attachment events, individuals, or feelings and is evidenced in story material that attempts to deflect and prevent the individual from becoming conscious of attendant attachment distress. Examples include references to social scripts (e.g., visiting a grave on the anniversary of the death), power (e.g., material wealth), achievement (e.g., intellectual pursuits), authority, or romance (diversion of attention from attachment needs to the sexual system). Deactivation is also evidenced by overt rejection, such as descriptions of characters as not deserving care, pushed away, or being punished for inconsequential transgressions. Cognitive disconnection, the splitting of attachment attention and affect, is evidenced when story elements are confusing or contradictory. Cognitive disconnection is also evidenced by themes of anger, withdrawal, or superficial sentimentality. Finally, segregated systems refer to the attempt to block painful distress, fear and rage, and are evidenced by descriptions of fear, isolation, abandonment, helplessness, and extreme vulnerability. “Normative” segregated systems are differentiated from “traumatic” forms. Normative segregated systems are story elements that have been empirically established as very common responses in community samples (e.g., fear of being hit by a ball while playing dodge ball). Traumatic segregated systems are defined as severe, eerie, evil, or surreal material (e.g., being beaten, disgust, rage, characters described as floating or as a statue – i.e., frozen). Traumatic segregated systems are also evident when individuals constrict and freeze in response to a stimulus, handing the picture back to the administrator or stating in a very firm way that they cannot provide a response. Pathological mourning for attachment trauma is designated when in a transcript three or more indicators of traumatic segregated systems are coded. Failed mourning for attachment trauma is designated when this pattern occurs for Ds cases. Preoccupation with attachment trauma is designated when this pattern occurs for E cases. Unresolved for attachment trauma is designated when this pattern occurs for U cases. This pattern is rare when the classification is F and in this case it is not considered as pathological mourning.

Two trained reliable AAP judges coded independently 20 randomly selected protocols. Cohen’s kappa on the AAP four-way classifications were *k* = 0.95. Final classifications regarding cases with disagreements between judges were determined by consensus. Inter-judge agreement for the pathological mourning attachment groups was *k* = 0.91, indicative of a nearly total agreement between observers (Viera and Garrett, 2005). The AAP demonstrated concurrent validity with the AAI (George and West, 2001; Buchheim and George, 2011). Predictive validity and interjudge reliability were demonstrated in a range of community and clinical studies (e.g., Béliveau and Moss, 2005; Van Ecke, 2005, 2006; Aikins et al., 2009; Webster et al., 2009).

## RESULTS

The first hypothesis was that the most prevalent attachment classifications in the current sample would be U and Ds. Results confirmed this expectation. The classification distribution is shown in **Table 1**. Twenty six (51%) patients were classified U, 19 (37%) patients were Ds, four were scored (8 %) E, and two patients (4%) were classified secure (F). In summary, 88% of the sample showed an unresolved or dismissing attachment pattern.

**Table 1:** Frequency and percentage of attachment pattern classification in Anorexia nervosa (AN) current patients (*n* = 51), van IJzendoorn and Bakermans-Kranenburg’s (1996) and Cassibba et al.’s (2013) samples.

	Current study		van IJzendoorn and Bakermans-Kranenburg (1996)				Cassibba et al. (2013)			
**Classification**	**AN female (*n*= 51)**		**Adolescent and young adults (*n*= 225)**		**Clinical sample (axis I and II) (*n*= 350)**		**Italian non-clinical adolescents (*n* = 336)**		Italian clinical sample (*n* = 260)	
	***n***	%	***n***	%	***n***	%	***n***	%	***n***	%
F	2	3.9	107	48	14	8	209	62	43	17
Ds	19	37.3	47	21	43	26	80	24	79	30
E	4	7.8	27	12	42	25	33	10	44	17
U	26	51.0	44	20	66	40	14	4	94	36

*F, Secure; Ds, Dismissing; E, preoccupied; U, Unresolved.*

In order to assess the second hypothesis, a series of Chi-square tests was performed to compare the current distribution to the community and clinical samples reported in van IJzendoorn and Bakermans-Kranenburg (1996) and Cassibba et al.’s (2013) meta-analyses.

Significant results were found for all comparisons (*p* < 0.05), suggesting that distributions were significantly different (**Table 2**). Patients with anorexia classified as unresolved in the current sample (60%) clearly outnumbered individuals with the same classification in both van IJzendoorn and Bakermans-Kranenburg (1996) as well as Cassibba et al.’s (2013) meta-analyses, in which the highest percentage of individuals scored as unresolved was 40% (van IJzendoorn and Bakermans-Kranenburg, 1996). In line with expectations, the percentage of dismissing classifications was higher in the current study than in both the above cited meta-analyses. Therefore, as expected, anorexic patients showed a significantly different distribution of attachment classifications in comparison to clinical samples recruited in previous studies.

**Table 2:** Chi-square test between the current sample (*n* = 51) and van IJzendoorn and Bakermans-Kranenburg’s (1996) and Cassibba et al.’s (2013) samples.

		χ^2^	*p*
van IJzendoorn and Bakermans-Kranenburg (1996)	Adolescent and young adults (225)	41.53	< 0.001
	Clinical sample (350)	9.57	0.023
Cassibba et al. (2013)	Italian non-clinical adolescents (336)	124.78	< 0.001
	Italian clinical sample (260)	10.04	0.018

Moving to the third aim, means and SDs of the AAP Deactivation and Segregated System processes are reported in **Table 3**. The mean of Deactivation defenses, in the overall sample, showed that patients used more than one Deactivation defense in each story (*M* = 9.54; DS = 7.22), while the mean of segregated systems (*M* = 7.53; SD = 4.58) showed that patients reported at least one segregated system in each story. The frequency of traumatic SS was significantly higher than the frequency of normative SS (Wilcoxon test, *z*= -1.74; *p* < 0.05, one tailed significance).

**Table 3:** Mean and SD of Attachment Projective Picture System (AAP) defensive processes according to the four ways AAP classification.

	AN female (*n*= 51)		F (*n*= 2)		Ds (*n*= 19)		E (*n*= 4)		U (*n*= 26)	
	*M*	SD	*M*	SD	*M*	SD	*M*	SD	*M*	SD
SS total	7.53	4.58	5.50	3.54	7.63	4.63	1.5	0.58	7.46	4.63
SS traumatic	4.69	4.76	1.50	2.14	4.42	4.76	.50	0.58	4.88	4.94
SS normative	2.84	2.86	4.00	5.66	3.21	3.38	1.00	0.82	2.58	2.45
Deactivation	9.54	7.22	7.00	1.41	12.47	7.48	4.25	2.63	7.23	6.18
Cognitive disconnection	16.27	9.26	22.00	2.83	19.32	10.38	7.00	2.45	14.04	7.83

*F, Secure; Ds, Dismissing; E, preoccupied; U, Unresolved; SS tot, Segregated System; SS traumatic, Segregated System Traumatic; SS normative, Segregated System Normative; Ds tot, Deactivation.*

The very limited number of F and E patients did not allow to pursue statistical analysis on these two categories of patients, thus the following statistical analyses were carried out only on patients classified as Ds or U. No significant differences were found between Ds and U patients as regards the frequency of total, normative, and traumatic segregated systems (Mann-Whitney test, respectively, *z* = -0.49, *p* = 0.62; *z* = -0.40, *p* = 0.69; *z* = -0.39, *p* = 0.69). Ds patients showed a significant higher frequency of Deactivation defenses than U patients (Wilcoxon test, *z*= -2.95; *p* < 0.01, one tailed significance).

The last set of analyses focused on pathological mourning. Overall, 26 patients (64.44%) showed a pathological mourning. Among them, 11 (37.9%) were classified as DS (failed mourning), while the remaining subjects (62.1%) were classified as U (unresolved for attachment trauma). Nineteen out of 45 (35.6%) patients showed only normative segregated systems (17.8%) or reported less than three traumatic segregated systems (17.8%).

A Chi-square test showed no significant differences in the distribution of pathological mourning versus normative mourning within the two considered attachment classifications (χ^2^_(1)_ = 0.62, *p* = 0.43).

## DISCUSSION

This study was firstly designed to replicate and extend existing literature concerning attachment classifications in anorexic patients. A second major aim was to provide an additional contribution to attachment-related issues in a sample of anorexic patients by means of a qualitative analysis and via the use of a conceptual model based on attachment trauma.

As expected, the distribution of attachment patterns in the current sample was significantly different from non-clinical samples (van IJzendoorn and Bakermans-Kranenburg, 1996; Cassibba et al., 2013), with an overrepresentation of the unresolved-disorganized attachment pattern, advocating for specificity in the link between attachment and AN.

The distribution of attachment patterns was in line with literature of clinical anorectic samples, showing that U and Ds attachment classifications were the most prevalent (Ramacciotti et al., 2001; Zachrisson and Kulbotten, 2006). More specifically, 37% of the sample was classified as Ds. These patients tend to maintain an avoidant, detached, or distanced position in relation to attachment. Such attitude implies the use of deactivation strategies in order to keep distressing emotions under control after attachment activation (George et al., unpublished manuscript). This dismissing attitude represents a defensive turning away from potentially painful emotional material, similar to the anorexic’s denial of hunger (Cole-Detke and Kobak, 1996; Dalgleish et al., 2001).

Most of AN patients (51%) were classified as U, confirming both their inability to use deactivation and/or cognitive disconnection strategies and to refer to internal working models of attachment in order to block attachment stress (Solomon et al., 1995; George and Solomon, 2008). Results also showed that they were significantly less able to use deactivation defenses than Ds patients. These findings, in line with previous studies, suggested that AN is associated with dismissing as well as unresolved-disorganized attachment (Fonagy et al., 1996; Ward et al., 2001; Ringer and Crittenden, 2007).

Moving to the third hypothesis, focused on the comparison between traumatic and normative segregated systems, findings showed that severe, eerie, evil, or surreal material in response to AAP stimuli (i.e., traumatic segregated systems) was significantly overrepresented when compared to common fearful, dangerous story elements (the normative segregated systems). No significant differences were found between Ds and U patients neither in traumatic nor in normative segregated systems. In other words, dismissing AN patients were overwhelmed by the same amount of traumatic and normative segregated systems, which, as previously mentioned, represent the main processes associated with unresolved attachment.

Focusing more on the use of segregated systems, the overrepresented presence of failed mourning (58%) in the Ds patients confirms the risk of a normative defense break-down due to dysregulated experience and affect. As a consequence, this leads to a disorganized form of chronic failed mourning (Bowlby, 1980). Similarly, the majority (69%) of U patients showed that normative defense break-down leads to a disorganized form of chronic pathological mourning (Bowlby, 1980). Qualitative analysis of the four patients classified as preoccupied confirmed the absence of pathological mourning.

Results of the present paper seem to support previous findings from neuropsychological studies on traumatic experience (Pearlman, 1995; Perry et al., 1995). Such research stressed how the unresolved attachment pattern could develop also through subtle and traumatic experiences of misattuned mothering, and not only via traumas-such as separation due to adoption, premature birth, or physical trauma. As a consequence of infants’ affective dysregulation experience, states of body-mind link disintegration occur. In such situations, the infant may attempt to find comfort by means of a permanent dissociation from intolerable levels of anxiety via self-regulating behaviors, such as thumb sucking or demands for food. These self-regulatory mechanisms may be imprinted as primary comfort and self-care, replacing early attachment relationships, and mutual caring. In addition, Schore (2000) and other neuropsychiatric researchers suggested that these dissociative processes could be biochemically imprinted in the brain. As Perry et al. (1995) suggested “states become traits.” Such psychobiological imprinting of early attachment disruptions would also explain why eating disorders are so often triggered by experiences of separation and loss or feared loss—puberty, leaving for college, breaking up with a boyfriend, or death of a loved one.

This study is not without limitations. First of all, the current study involved a small sample of anorexic girls who were in-patients in treatment at the time of the study, which underlines their severe health conditions. Thus, results from the current work may not be representative of the general anorexic population. Second, the AAP, differently from the AAI, does not give any autobiographical information about patients. In order to have a deeper knowledge of patients’ personal story, future studies should use either AAP, and AAI together, or AAP and other measures to assesses traumatic experiences and pathological mourning. Third, this study lacks information about the relationship between symptom severity and attachment. Last but not least, the present research did not involve a community-based control group sample or other psychiatric groups diagnosed with different eating disorders for comparison. Finally, our data do not address issues of causality. Future studies should use longitudinal prospective designs as to assess attachment strategies across time, starting before the emergence of any symptoms.

However, beside these limitations, the AAP, compared with the AAI, proved itself a useful tool in the evaluation of unconscious defensive processes and in the identification of segregated systems. It allowed the examination of new and unique construct-based features of attachment, overcoming limits of the AAI. The issue of unresolved attachment and of its internalization, via the analysis of the internal quality of segregated systems across AAP stories, was of interest as to understand the presence and the quality of pervasive dysregulation mechanisms and pathological mourning in AN patients. This issue has never been studied before in eating disorder patients, which highlights the importance of the present work. Given the interesting findings from this study, our model could be considered as a pilot framework for future research employing larger samples of eating disorder patients.

The developmental perspective in attachment research seems to offer a helpful contribution also to the clinical field, suggesting useful criteria for the choice of suitable treatment interventions with AN patients. Given the pilot nature of this study, we expect to expand these findings to get to a better understanding of the current data. Moreover, given the paucity of studies which have so far applied the AAP to analyze psychological functioning in AN patients, we hope that these pilot results will provide some valid reasons for improving the heuristic power of this method.

## Conflict of Interest Statement

The authors declare that the research was conducted in the absence of any commercial or financial relationships that could be construed as a potential conflict of interest.
